# Specific egg yolk immunoglobulin as a promising non-antibiotic biotherapeutic product against *Acinetobacter baumannii* pneumonia infection

**DOI:** 10.1038/s41598-021-81356-8

**Published:** 2021-01-21

**Authors:** Abolfazl Jahangiri, Parviz Owlia, Iraj Rasooli, Jafar Salimian, Ehsan Derakhshanifar, Zahra Aghajani, Sajad Abdollahi, Saeed Khalili, Daryush Talei, Elham Darzi Eslam

**Affiliations:** 1 Department of Biology, Shahed University, Tehran-Qom Express Way, 3319118651 Tehran, Iran; 2 Applied Microbiology Research Center, Systems Biology and Poisonings Institute, Baqiyatallah University of Medical Sciences, Tehran, Iran; 3 Molecular Microbiology Research Center and Department of Biology, Shahed University, Tehran, Iran; 4 Department of Microbiology, Faculty of Medical Sciences, Shahed University, Tehran, Iran; 5 Chemical Injuries Research Center, Systems Biology and Poisonings Institute, Baqiyatallah University of Medical Sciences, Tehran, Iran; 6 Ph.D. Student of Medical Biotechnology, Hamadan University of Medical Sciences, Hamadan, Iran; 7 Behbahan Khatam Alanbia University of Technology, Behbahan, Iran; 8 Department of Biology Sciences, Shahid Rajaee Teacher Training University, Tehran, Iran; 9 Medicinal Plants Research Center, Shahed University, Tehran, Iran

**Keywords:** Bacterial infection, Protein vaccines, Immunology, Microbiology

## Abstract

*Acinetobacter baumannii* is a serious health threat with a high mortality rate. We have already reported prophylactic effects of IgYs raised against OmpA and Omp34 as well as against inactivated whole-cell (IWC) of *A. baumannii* in a murine pneumonia model. However, the infection was exacerbated in the mice group that received IgYs raised against the combination of OmpA and Omp34. The current study was conducted to propose reasons for the observed antibody-dependent enhancement (ADE) in addition to the therapeutic effect of specific IgYs in the murine pneumonia model. This phenomenon was hypothetically attributed to topologically inaccessible similar epitopes of OmpA and Omp34 sharing similarity with peptides of mice proteins. In silico analyses revealed that some inaccessible peptides of OmpA shared similarity with peptides of Omp34 and *Mus musculus*. Specific anti-OmpA and anti-Omp34 IgYs cross-reacted with Omp34 and OmpA respectively. Specific IgYs showed different protectivity against *A. baumannii* AbI101 in the murine pneumonia model. IgYs triggered against OmpA or IWC of *A. baumannii* were the most protective antibodies. IgY triggered against Omp34 is ranked next after those against OmpA. The lowest protection was observed in mice received IgYs raised against the combination of rOmpA and rOmp34. In conclusion, specific IgYs against OmpA, Omp34, and IWC of *A. baumannii* could serve as novel biotherapeutics against *A. baumannii* pneumonia.

## Introduction

*Acinetobacter baumannii* is a emerged serious health threat^1^. World Health Organisation (WHO) has assigned this pathogen as the priority that needs new antibiotics^2^. This successful nosocomial pathogen is considered by the Infectious Diseases Society of America (IDSA) as one of the six most dangerous microbes^3^. High mortality rate (more than 70%)^4^ and difficulty in the clinical management of *A. baumannii*-associated infections are attributed to the emergence of highly antibiotic-resistant strains^5–7^. These implications motivated researchers to introduce efficient antibiotics against this notorious pathogen. However, no efficient antibiotic is provided so far by the pharmaceutical industry^7^. Active and passive immunizations have been considered as alternative cost-effective treatment options^8^. In this regard, passive immunization using specific antibodies is a promising strategy against infections caused by *A. baumannii*^3,8–12^. Avian egg yolk IgY, a counterpart immunoglobulin to mammalian IgG, has several advantages to be considered suitable for passive immunization. It is a cost-effective natural product that does not activate the complement system. Production of IgY is not invasive since no bleeding is needed. Moreover, a high amount of specific IgY could be recovered from the egg yolk of immunized hens^13,14^. Anti-*Pseudomonas* IgY has been successfully applied in clinical trials^15^. This specific IgY could modify bacterial fitness, increase bacterial hydrophobicity, and facilitate the formation of immobilized bacteria in aggregates. It could enhance bacterial killing via Polymorphonuclear neutrophils (PMN)^16,17^. The anti-*P. aeruginosa* IgY could also significantly reduce the bacterial burden and inflammatory cytokines in the lung of a murine (BALB/c) pneumonia model^18^. Protective effect of specific anti-*Acinetobacter* IgYs raised against OmpA, Omp34 (also known as Omp34kDa or Omp33-36), or inactivated the whole-cell of *A. baumannii* ATCC 19606 was demonstrated in our recent study^19^. OmpA and Omp34 are major virulence factors of *A. baumannii* involved in the bacterial adhesion to the human lung epithelial cell line i.e. A549 cells^20^. These virulence factors are highly immunogenic proteins^10,21–23^. OmpA, one of the most abundant OMP of the pathogen^24^, is the most promising *A. baumannii* immunogen amongst introduced protein antigens^25^. Omp34 is a specific antigen suggested to be appropriate for diagnostic kits development^23^. This OMP is a 14-stranded barrel^26^ harboring loops sharing similarity with human peptides^27^.

Despite higher absorbance in ELISA, specific IgY triggered against the combination of OmpA and Omp34 unexpectedly exacerbated disease in comparison to other IgYs^19^. In addition to the study, which was the first report on antibody-dependent enhancement (ADE) of *A. baumannii* infection, recently, this phenomenon has also been reported on a mAb developed against K2 capsular polysaccharide of *A. baumannii*^28^. The study suggests that the capsule could act as decoys interacting with the mAb^28^. Administration of anti-*Acinetobacter* IgYs before the challenge with viable pathogens could cause protection. However, the post-challenge administration of the IgY needs to be investigated. The occurrence in the murine pneumonia model of ADE is not clear. This phenomenon was attributed to the existence of similar epitopes within OmpA and Omp34 topologically are inaccessible^19^. As a complementary hypothesis, some of these inaccessible epitopes of OmpA and Omp34 are similar to the epitopes of mice proteins. The present study is designed to evaluate the therapeutic effects of specific IgYs against *A. baumannii* infection in a murine pneumonia model. The abovementioned hypothesis is also analyzed by an in silico approach. Also, cross-reaction of the specific IgYs with each other was assessed by immunoassay tests. The results of this study could elaborate the role of IgYs as novel efficient treatment options to apply against *A. baumannii* infections. To the best of our knowledge, this is the first report on the therapeutic effects of the specific IgYs against *A. baumannii*.

## Materials and methods

### In silico analyses

In silico analyses were performed on the probable similarity of some of the inaccessible epitopes of OmpA and Omp34 with the epitopes of mice proteins. The sequences of OmpA and Omp34^26,27,29^ were aligned by PRALINE^30^ at http://www.ibi.vu.nl/programs/pralinewww/. The primary sequence similarity was carried out as follows: the mature Omp34 sequence was spliced to 12-residue peptides offset by 3 amino acids; the obtained peptides were searched within the OmpA sequence via Epitope Conservancy Analysis tool at http://tools.iedb.org/conservancy/. The peptides (from Omp34 and OmpA) sharing > 40% identity were subjected to a BLAST search (with ≥ 70% identity) at http://www.iedb.org/ to find similar linear epitopes in *Mus musculus*. The topology of the peptides was elucidated from our previous in silico studies^26,27,29^.

### Immunoassay tests

The IgYs were prepared as described earlier^19^. Western blotting was carried out as follows: the recombinant proteins, rOmpA, and rOmp34 were loaded on 8% SDS-PAGE. The proteins were then transferred onto a nitrocellulose membrane via electro-blotting at 75 V for 2 h followed by blocking of the membrane with 5% skimmed milk in phosphate-buffered saline with Tween-20 (PBST) with gentle shaking overnight at 4 °C. The membrane was washed thrice with PBST and was then incubated with specific IgYs raised against rOmpA, rOmp34, or Inactivated Whole Cell, with gentle shaking for 2 h. A dilution of IgYs at OD_450_ of ~ 1.5 that was already observed in indirect ELISA^19^ was used. The specific IgYs were diluted in PBST. The washing step was repeated and the membrane was then submerged in HRP-conjugated rabbit anti-chicken IgG antibody (Sigma-Aldrich) (1:2000 diluted in PBST) with gentle shaking for 2 h.

Sets of indirect ELISA were harnessed to assess the immunoreactivity and cross-reactivity of IgYs. The reactivity of the IgYs was initially evaluated against the whole cell of *A. baumannii* ATCC 19,606. The wells were coated with *A. baumannii* at a concentration of 3.6 × 10^7^ CFU/well suspended in 50 mM sodium carbonate buffer, pH 9.6, and were then incubated at 4 °C overnight. The wells were washed 3 times with PBS containing 0.05% Tween 20 (PBST). Blocking buffer (100 μL of PBST containing 5% skim milk) was added to the wells followed by incubation at 37 °C for 1 h. The wells were washed thrice with PBST followed by the addition of 100 μl specific IgY solutions prepared in PBS (5 and 2.5 µg/well). The plate was then incubated for 1 h at 37 °C. The washing procedure was repeated and 100 μL of HRP-conjugated rabbit anti-chicken IgG antibody (Sigma-Aldrich) diluted to 1:1500 in PBST was added to the wells. The plate was incubated at 37 °C for 1 h, washed with PBST 3 times followed by the addition of 100 μL of TMB solution. The plate was kept in dark at room temperature for 15 min to develop a minor color change in control wells. 100 μl of 3 M H_2_SO_4_ was added to each well to stop the reaction. The optical density was measured at 450 nm on an ELISA reader. Another ELISA test was designed to assess the reactivity of specific IgY-IWC (IgY raised against Inactivated Whole-Cell) against recombinant OmpA and Omp34 proteins. The ELISA was performed as mentioned above except for the antigen coating step. 2 µg of the recombinant OmpA or Omp34 suspended in 50 mM sodium carbonate buffer, pH 9.6 was coated in each well. To validate the synergic effect of the combination of rOmpA and rOmp34 as antigens administered for hen immunization, an ELISA test was performed. In this ELISA, rOmpA or rOmp34 (2 µg/well) were coated as antigens. IgYs (0.625, 0.312 or 0.157 µg/well) added to the wells were as follow: IgY-A (IgY raised against rOmpA), IgY-34 (IgY raised against rOmp34), IgY-A + 34 (IgY raised against the combination of rOmpA and rOmp34) or IgY-mix (a mixture of IgY-A and IgY-34 in 1:1 ratio). The last ELISA was carried out to assess cross-reactivity of the IgY-A and IgY-34 to rOmp34 and rOmpA respectively.

### Challenge of mice

The mice were placed in the animal house at Shahed University under standard conditions and the research was conducted following the principles offered in the Guide for the Care and Use of laboratory animals (NIH Publications no. 8023, revised 1978). Ethical approval was issued by ethical committee of Shahed University. The animals were maintained in a well-ventilated environment with water and animal feed ad libitum. The study was carried out in compliance with the ARRIVE guidelines.

#### Pilot challenges with *A. baumannii* ATCC 19606

*A. baumannii* ATCC 19606 was used as a standard strain in pilot challenges. Pilot challenges were carried out to estimate appropriate doses of effective IgYs. For this purpose, only IgY-IWC and IgY-C (control IgY) were used. To determine 50% lethal dose (LD_50_) of *A. baumannii* ATCC 19606 in the murine pneumonia model, four groups of BALB/c mice (six mice per group) received intraperitoneally Cyclophosphamide (150 µg/g) on days 1 and 2. On the fourth day, 40 µl of bacterial suspensions in PBS (ranging from 1.8 × l0^7^ to 1.8 × l0^9^ CFU) were administrated intranasally. The LD_50_ was estimated by the Probit method^31^ based on the number of survivors 3 days after the challenges. On days 1 and 2, mice groups (five groups of 27–32 g BALB/c mice) received an intraperitoneal dose of 150 µg/g of Cyclophosphamide. On day 4, the mice were anesthetized with an intraperitoneal injection of Xylazine (20 mg/kg) + Ketamine (100 mg/kg) and were intranasally challenged with ~ 1.8 × 10^9^ CFU of *A. baumannii* ATCC 19,606. Four hours after the challenge, 20 µl of PBS or IgYs were intranasally administered. The control group received sterile PBS. The remaining groups received 40 or 100 µg of IgYs (IgY-IWC or IgY-C). Mice were monitored for 14 days after which, the survivors were euthanized. Their spleens and lungs were aseptically excised, weighed, and homogenized in 0.9% sterile NaCl. Serial dilutions were made from the homogenates and 100 µl from each dilution was cultured on LB agar medium. The plates were incubated overnight at 37 °C for colony counts.

#### Clinical strain

For a challenge with clinical strain, AbI101 nominated as the most fatal among the six evaluated strains^19^, was selected. The bacterial LD_50_ was determined in the murine pneumonia model. In this regard, five groups (n = 6 mice/group) of neutropenic BALB/c mice were studied. 20 µl of bacterial suspensions in PBS ranging from 1.18 × l0^6^ to 6 × l0^8^ CFU was intranasally administrated. The LD_50_ was estimated by the Probit method^31^ based on the number of survivors 3 days post-challenge. Eight groups of BALB/c mice were considered for the challenge. Five groups received 40 µg of IgY-C, IgY-A, IgY-34, IgY-A + 34, or IgY-IWC 4 h after challenge with 5.65 × 10^8^ CFU of *A. baumannii* AbI101. One group received 80 µg of IgY-A + 34 4 h after challenge with 5.65 × 10^8^ CFU of the bacteria. Two control groups only received bacteria or IgY. All administrations were carried out via the intranasal route. The mice were monitored for 8 days. The survivors were euthanized and their spleens and lungs were aseptically excised, weighed, and homogenized in 0.9% sterile NaCl. 100 µl from each tissue dilutions was plated on LB agar medium, incubated at 37 °C overnight to determine CFU/ Tissue. In addition to survival monitoring, clinical symptoms (weight loss, piloerection, clustering, hypothermia and tachypnea) as well as eye condition (a range between no sign and closed septic eyes) of the mice were also considered.

### Statistical analyses

Data were subjected to analysis of variance and Kruskal–Wallis test using SPSS25. Duncan’s multiple range and non-parametric log-rank tests were performed for comparison of means at *P* ≤ 0.05. The graph pad prism software version 8 was used for drawing the graphs.

## Results

### Similarity sharing of inaccessible peptides of OmpA with the peptides of Omp34 and *Mus musculus*

The protein sequences of OmpA and Omp34 shared a 10% sequence identity. Peptides of Omp34 were aligned with OmpA to find potential epitope similarity. Twenty-one 12-meric peptides shared > 40% identity with OmpA peptides. To find similar linear epitopes in *Mus musculus,* these peptides served as queries in BLAST searches. None of the Omp34-derived peptides matched with linear epitopes of *Mus musculus*. However, two topologically inaccessible peptides (LSLRTEARATYN and PVEPTPVAPQPQ) of OmpA shared similarity with 2 and 18 linear epitope identifiers (IDs) of *Mus musculus* respectively (Table 1). These murine epitopes were of CD180 antigen, Myelin-associated neurite-outgrowth inhibitor, Hematological and neurological expressed 1 protein and Tyrosine-protein phosphatase non-receptor type 23. Table 2 shows the topology of OmpA- and Omp34-derived peptides.

**Table 1:** Similar peptides of Omp34and OmpA.

Peptide sequence of Omp34	Position on Omp34	Peptide sequence of OmpA	Position on OmpA	Identity (%)	Similar mice peptide to Omp34 peptide	Similar mice peptide to OmpA peptide
GQSEYVDTTAND	26–37	KL**SEY**PNA**TA**RI	255–266	41.67	Not found	Not found
ANDKNFTGDVAG	35–46	**A**EYNQVK**GDV**D**G**	77–88	41.67	Not found	Not found
EAAFLNQASSVS	62–73	**E**RLS**L**AR**A**N**SV**K	280–291	41.67	Not found	Not found
SVSLGYSYQQYD	71–82	Y**V**L**LG**AGHYK**YD**	122–133	41.67	Not found	Not found
LGYSYQQYDQNN	74–85	**LGY**TF**Q**DSQH**NN**	31–42	50.00	Not found	Not found
PYLPVYASATYN	104–115	LS**L**RTE**A**R**ATYN**	165–176	50.00	Not found	LSYNEPLSLKTEA LSYNEPLSLKTEAF
PVYASATYNHTD	107–118	RTE**A**R**ATYN**ADE	168–179	41.67	Not found	Not found
ASATYNHTDVDG	110–121	**A**EYNQVKG**DVDG**, **A**R**ATYN**ADEEFW, K**SA**LV**N**EYN**VD**A	77–88, 171–182, 291–302	41.67	Not found	Not found
HTDVDGKNNFSK	116–127	TS**D**LIT**KN**YD**SK**	107–118	41.67	Not found	Not found
VDGKNNFSKDDN	119–130	DSQH**NN**GG**KD**G**N**	37–48	41.67	Not found	Not found
KNNFSKDDNGDR	122–133	H**NN**GG**KD**G**N**LTN	40–51	41.67	Not found	Not found
GDRYALEVGAML	131–142	ELQDD**L**F**VGA**A**L**	54–65	41.67	Not found	Not found
YALEVGAMLLPN	134–145	DD**L**F**VGA**A**L**GIE	57–68	41.67	Not found	Not found
TVGYTSVANQFA	149–160	P**V**EP**T**P**VA**P**Q**PQ	209–220	41.67	Not found	18 peptides*
NQTAAIQNDQDA	176–187	**N**A**TA**R**I**EGHT**D**N	261–272	41.67	Not found	Not found
GFEAAGAFGQEN	206–217	**G**NAGV**GAF**WRL**N**	151–162	41.67	Not found	Not found
DLYLTPKLSVGA	224–235	GPE**L**QDD**L**F**VGA**, GIE**LTP**W**L**GFE**A**	52–63, 66–77	41.67	Not found	Not found
LTPKLSVGATFV	227–238	**L**QDD**L**F**VGA**ALG, **LTP**W**L**GFE**A**EYN	55–66, 69–80	41.67	Not found	Not found
KLSVGATFVGND	230–241	NAG**VGA**FWRL**ND**	152–163	41.67	Not found	Not found
VGATFVGNDGEA	233–244	**VGA**FWRL**ND**ALS	155–166	41.67	Not found	Not found
ITPALAVGASYM	266–277	L**TP**W**L**GFE**A**E**Y**N	69–80	41.67	Not found	Not found

Bold letters of OmpA peptides are identical residues of OmpA and Omp34 peptides.

Peptides of Omp34and OmpA shared similarity with linear epitopes of *Mus musculus* are shown separately.

The bold amino acid letters are identical in OmpA and Omp34 sequences.

*SPTPVAPHPVTVPT, TPVAPHPVTVPT, EKPVPAAPVPSPVAPAP, EKPVPAAPVPSPVAPAPVP, KPVPAAPVPSPVAPAPVP, APISSHTAPRPNPTPALPQP, APVPSPVAPAPVPSR, APVPSPVAPAPVPSRRNPPG, EEKPVPAAPVPSPVAPAPV, EEKPVPAAPVPSPVAPAPVP, EEKPVPAAPVPSPVAPAPVPS, EKPVPAAPVPSPVAPA, EKPVPAAPVPSPVAPAPV, EKPVPAAPVPSPVAPAPVPS, KPVPAAPVPSPVAPA, KPVPAAPVPSPVAPAP, KPVPAAPVPSPVAPAPV and KPVPAAPVPSPVAPAPVPS.

**Table 2:** Topology of the similar OmpA and Omp34 peptides.

Peptide sequence	Topology on Omp34	Peptide sequence on OmpA	Topology on OmpA
GQSEYVDTTAND	TM1, L1	KL**SEY**PNA**TA**RI	periplasmic
ANDKNFTGDVAG	L1	**A**EYNQVKGDVDG	TM3, L2
EAAFLNQASSVS	TM2, In1, TM3	**E**RLS**L**AR**A**N**SV**K	periplasmic
SVSLGYSYQQYD	TM3, L2	Y**V**L**LG**AGHYKYD	TM5, L3
LGYSYQQYDQNN	TM3, L2	**LGY**TF**QDSQHNN**	TM1, L1
PYLPVYASATYN	In2, TM5	LS**L**RTE**A**R**ATYN**	TM7
PVYASATYNHTD	TM5, L3	RTE**A**R**ATYNADE**	TM7, L4
ASATYNHTDVDG	TM5, L3	**A**EYNQVKGDVDG, **A**R**ATYNADEEFW**, K**SA**LV**N**EYN**VD**A	TM3, L2 TM7, L4 periplasmic
HTDVDGKNNFSK	L3	TS**D**LIT**KN**YD**SK**	TM4, In3, TM5
VDGKNNFSKDDN	L3	DSQHNNGGKDGN	L1
KNNFSKDDNGDR	L3	HNNGGKDGNLTN	L1
GDRYALEVGAML	L3, TM6	ELQDDLF**VGA**A**L**	L1, TM2
YALEVGAMLLPN	TM6, In3	DDLF**VGA**A**L**GIE	L1, TM2
TVGYTSVANQFA	TM7, L4	P**V**EP**T**P**VA**P**Q**PQ	periplasmic
NQTAAIQNDQDA	L4	**N**A**TA**R**I**EGHT**D**N	periplasmic
GFEAAGAFGQEN	TM9, L5	**G**NAGV**GAF**WRL**N**	TM6
DLYLTPKLSVGA	TM10, In5, TM11	GPELQDDLF**VGA**, GIE**LTP**W**L**GFE**A**	L1, TM2 TM2, In2, TM3
LTPKLSVGATFV	In5, TM11	**LQDDL**F**VGA**ALG, **LTP**W**L**GFE**A**EYN	L1, TM2 In2, TM3
KLSVGATFVGND	TM11, L6	NAG**VGA**FWRL**ND**	TM6, In4
VGATFVGNDGEA	TM11, L6	**VGA**FWRL**ND**ALS	TM6, In4
ITPALAVGASYM	In6, TM13	L**TP**W**L**GFE**A**E**Y**N	In2, TM3

Exposed residues located in external loops are underlined.

*TM* transmembrane, *In* internal turns, *L* external loops.

### Cross reaction of the specific anti-OmpA and anti-Omp34 IgYs with Omp34 and OmpA

The reactivity of the specific IgYs was evaluated by western blotting and various ELISAs. In the performed western blotting, IgY-A and IgY-34 showed significant reactivity with their corresponding antigens (rOmpA and rOmp34 respectively). IgY-IWC recognized both recombinant OMPs and developed sharp bands. IgY-A and IgY-34 showed cross-reactivity with rOmp34 and rOmpA respectively (see Supplementary Fig. S1).

**Figure 1:**
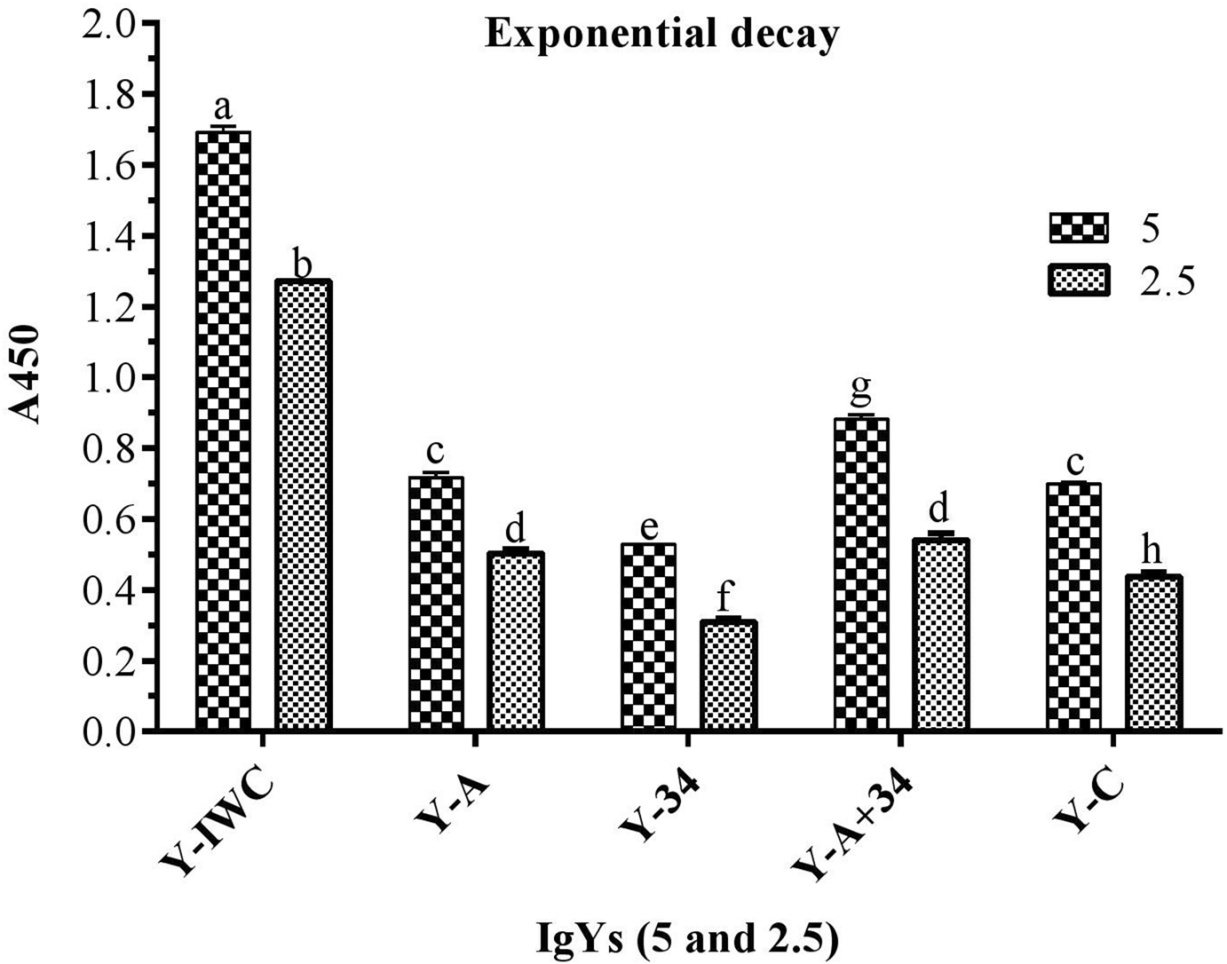
Whole cell ELISA with various IgYs. *A. baumannii* ATCC 19606 was coated as an antigen (3.6 × 10^7^ CFU/well); 5 µg and 2.5 µg of each IgY was added to the wells. Y-IWC: IgYs raised against inactivated whole cell, Y-A: IgYs raised against rOmpA, Y-34: IgYs raised against rOmp34, Y-A + 34: IgYs raised against combination of rOmpA and rOmp34; and Y-C: control IgY. Mean values ± S.E are from independent groups and values superscripted by different letters are significantly different by Duncan’s multiple range tests (*P* ≤ 0.01).

**Figure 2:**
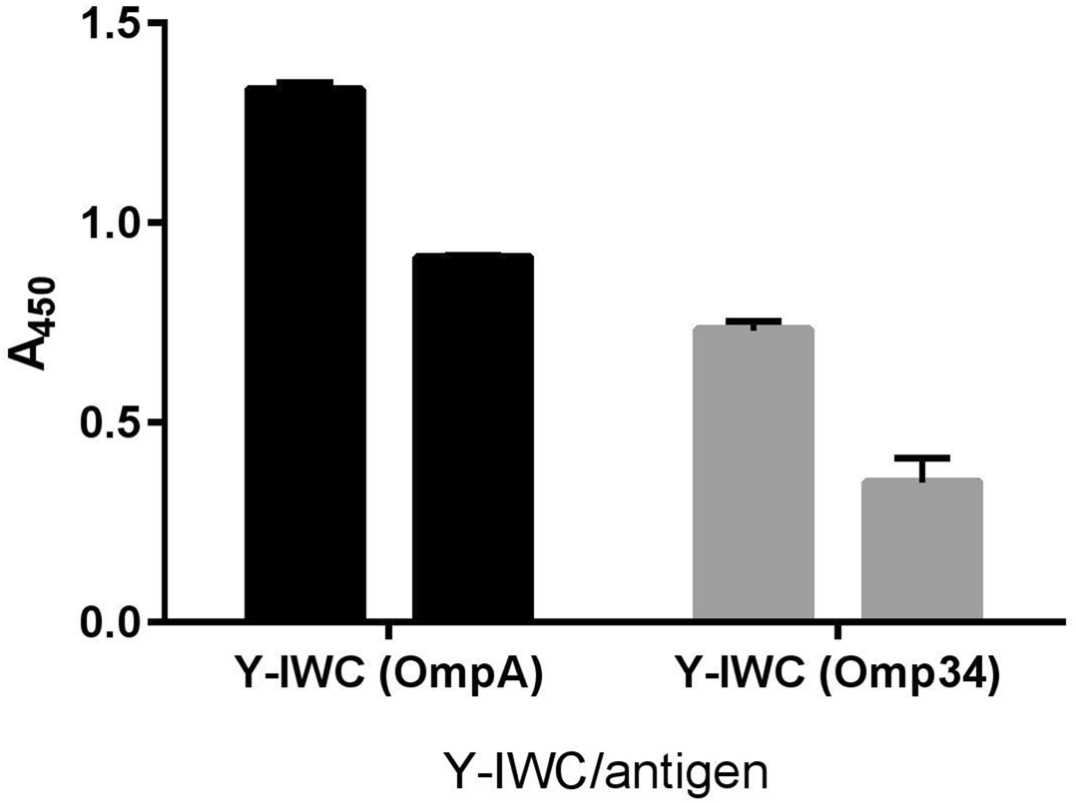
ELISA of rOmpA and rOmp34 antigens with IgY-IWC (Y-IWC). Two micrograms of antigens were coated. OmpA and Omp34 in parentheses show coated antigens. 5 and 2.5 µg of IgY-IWC was added to the wells. Y-IWC: IgYs raised against inactivated whole cell; the absorbance difference between rOmpA and rOmp34 antigens was significant using t-test (*P* ≤ 0.01).

**Figure 3:**
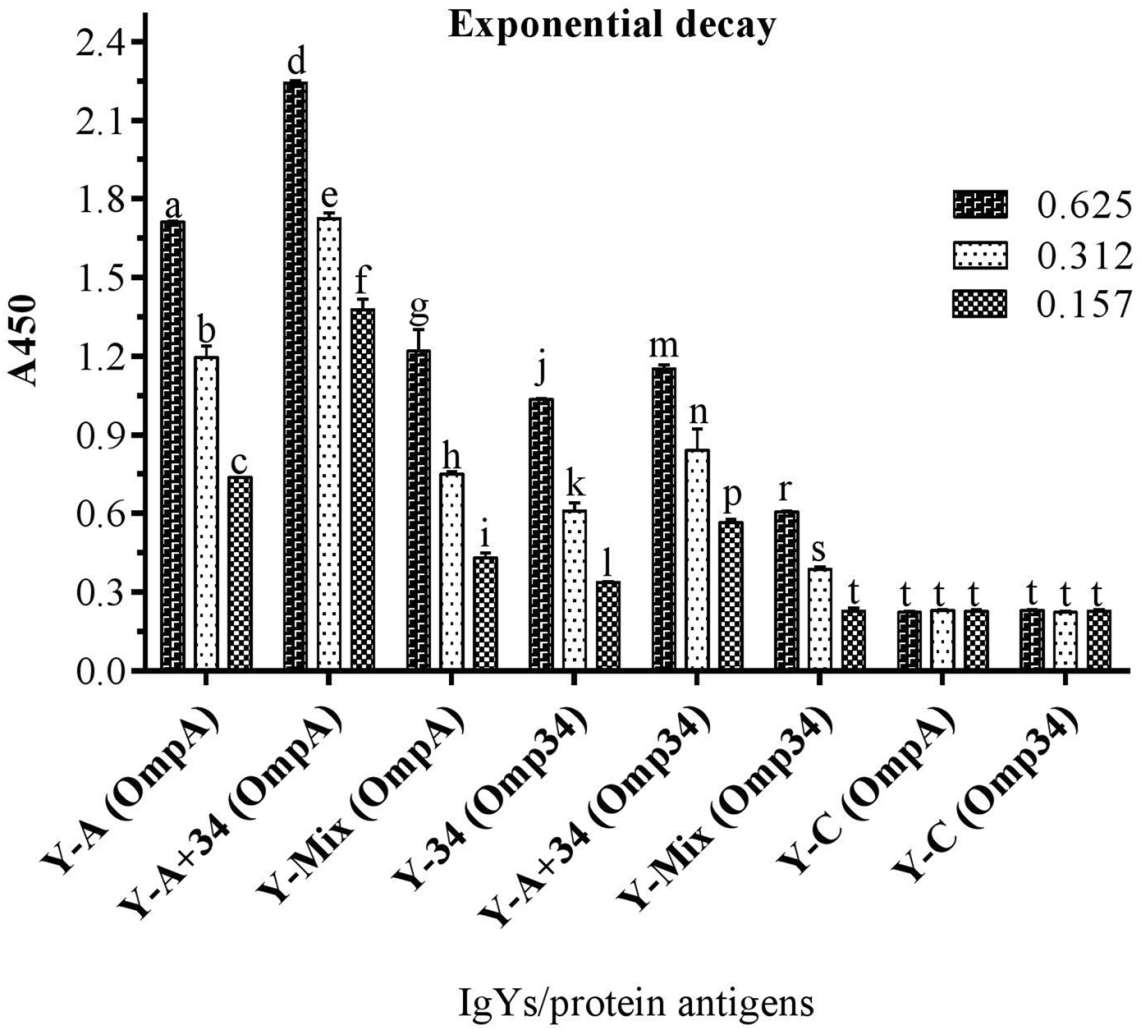
ELISA of specific IgYs against recombinant proteins to assay synergic effect of the antigens. Two micrograms of antigens were coated. OmpA and Omp34 in parentheses show coated antigens. Three levels of IgYs (0.625, 0.312 or 0.157 µg/well) were assessed. Y-A: IgYs raised against rOmpA, Y-34: IgYs raised against rOmp34, Y-A + 34: IgYs raised against combination of rOmpA34 and rOmp34 in 1:1 ratio, Y-Mix: mixture of Y-A and Y-34 in 1:1 ratio, and Y-C: control IgY. Mean values ± S.E are from independent groups and values superscripted by different letters are significantly different by Duncan’s multiple range tests (*P* ≤ 0.01).

**Figure 4:**
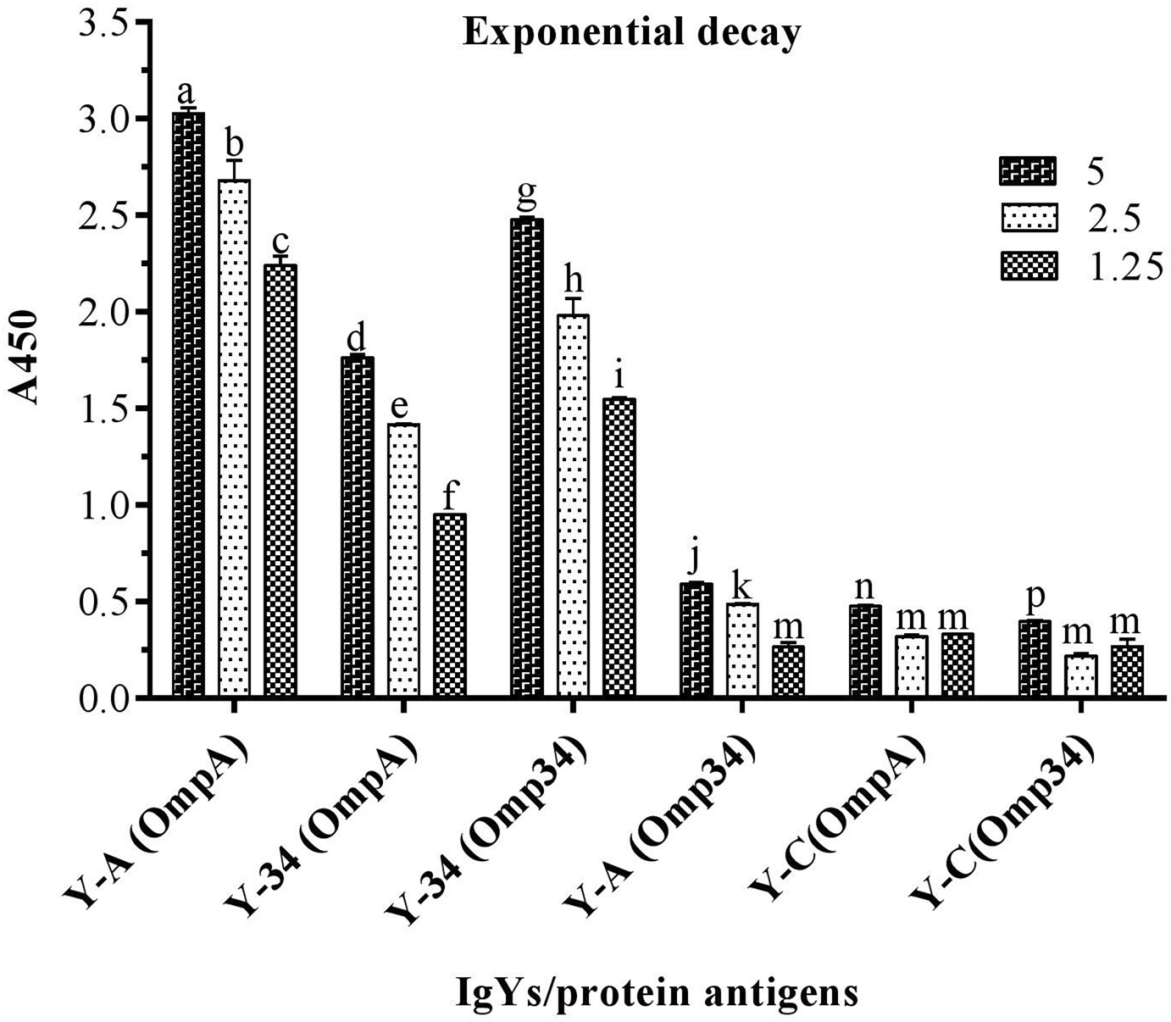
ELISA for cross-reactivity evaluation. Two micrograms of antigens was coated. Three levels of IgYs (5, 2.5 or 1.25 µg/well) were assessed. OmpA and Omp34 in parentheses show coated antigens. Y-A: IgYs raised against rOmpA. Y-34: IgYs raised against rOmp34. Mean values ± SE are from independent groups and values superscripted by different letters are significantly different as determined by Duncan’s multiple range tests (*P* ≤ 0.01).

In the ELISA against the whole cell of *A. baumannii* ATCC 19,606, all the specific IgYs detected WC in IgY-IWC > IgY-A + 34 > IgY-A > IgY-34 order. Control IgY (IgY-C), showed absorbance near to those obtained for IgY-A (Fig. 1). In ELISA performed against the recombinant proteins (rOmpA and rOmp34), IgY-IWC detected both recombinant proteins. However, absorbance in wells coated with rOmpA was approximately twofold higher than those coated with rOmp34 (Fig. 2). The results of ELISA carried out to evaluate the synergic effect of rOmpA and rOmp34 antigens in hen immunization showed that the absorbance of IgY-A + 34 was significantly higher than IgY-mix (Fig. 3). The fourth set of ELISA showed that IgY-34 could recognize rOmpA and vice versa. However, cross-reactivity of IgY-34 with rOmpA is about threefold higher than cross-reactivity of IgY-A with rOmp34 (p < 0.002) (Fig. 4).

### Challenges

#### Effect of the various quantities IgYs

**Figure 5:**
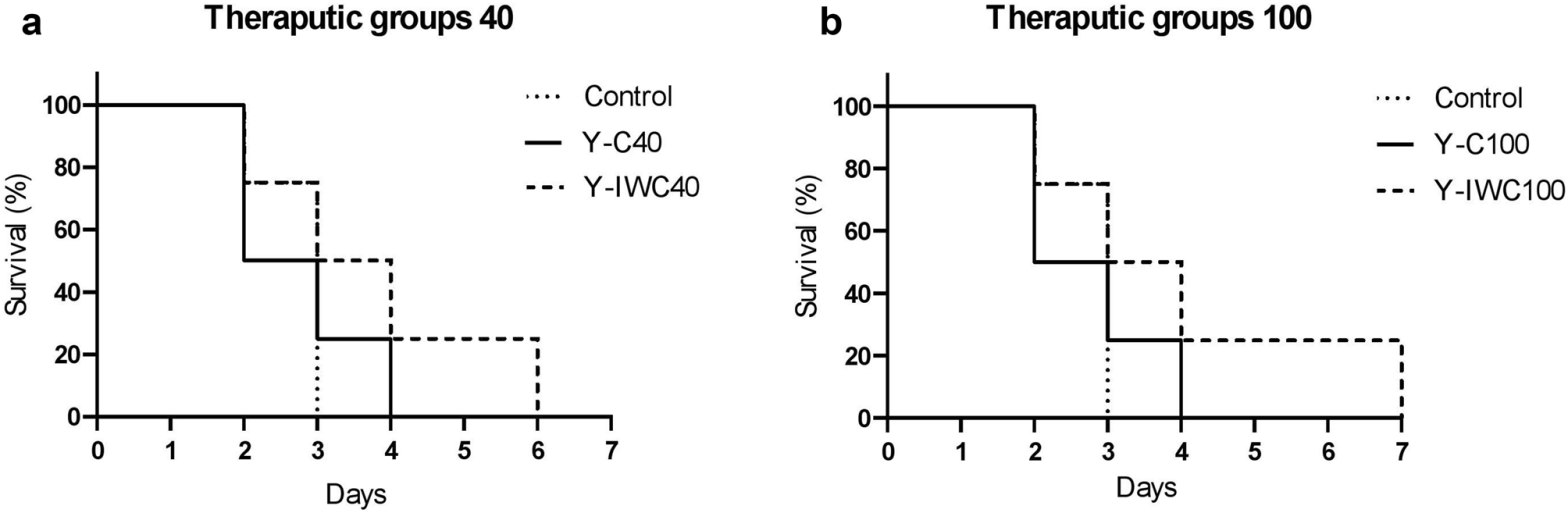
Survival plots of pilot mice groups. Mice groups received 40 µg (**a**, Y-C40 and Y-IWC40) or 100 µg (**b**, Y-C100 and Y-IWC100) of IgYs 4 h post-challenge with *A. baumannii* ATCC 19,606. Y-C: IgY-C, control IgY; Y-IWC: IgY-IWC, IgY raised against Inactivated Whole Cell.

To arrive at the appropriate effective doses of IgYs, a pilot challenge was carried out in which *A. baumannii* ATCC 19,606 was used as the standard strain. The mice were neutropenized with an intraperitoneal injection of cyclophosphamide. Pneumonia was developed by the nasal administration of *A. baumannii*. The LD_50_ of *A. baumannii* ATCC 19,606 was determined at 1.75 × 10^8^ CFU. The pilot mice groups received 40 µg or 100 µg of IgYs (IgY-C or IgY-IWC) 4 h post-challenge with *A. baumannii* ATCC 19,606. The mice challenged with 10 × LD_50_ showed clinical signs such as eye infection, weight loss, piloerection, clustering, hypothermia, and tachypnea. Survival was compared using the non-parametric log-rank test. No significant difference (p > 0.05) was seen between mice groups receiving 40 µg or 100 µg of specific IgYs (Fig. 5).

#### Protective effects of the specific IgYs against *A. baumannii* AbI101 in the murine pneumonia model

**Figure 6:**
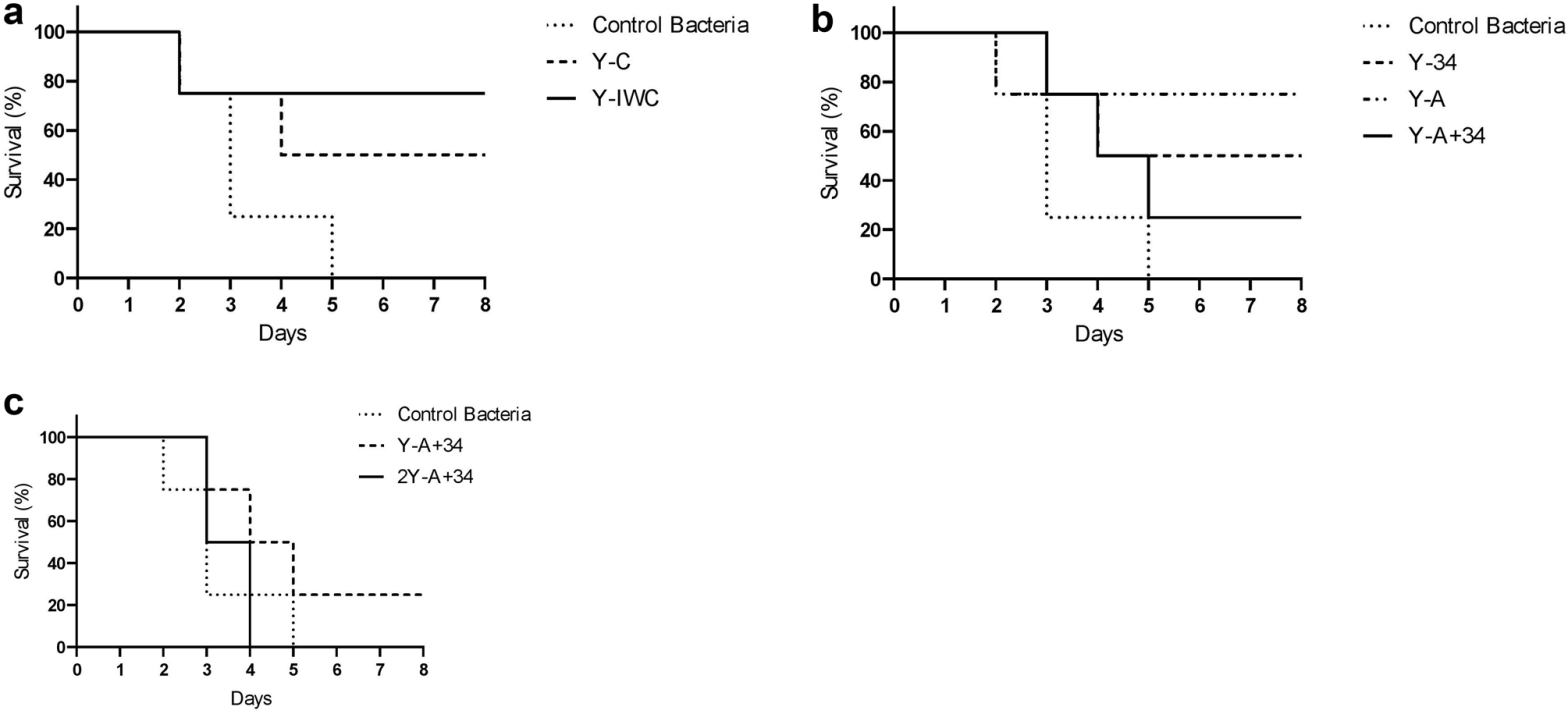
Survival plots of mice treated with specific IgYs 4 h after challenge with *A.baumannii* AbI101 suspended in PBS compared to the control group receiving 5.65 × 10^8^ CFU of *A. baumannii* AbI101. **a** Y-C: Mice receiving 40 µg of IgY-C after challenge with the bacteria. Y-IWC: Mice receiving 40 µg of IgY-IWC after challenge with the bacteria. **b** Y-34: Mice receiving 40 µg of IgY-34 after challenge with the bacteria. Y-A: Mice receiving 40 µg of IgY-A after challenge with the bacteria. Y-A + 34: Mice receiving 40 µg of IgY-A + 34 after challenge with the bacteria. **c** Y-A + 34: Mice receiving 40 µg of IgY-A + 34 after challenge with the bacteria. 2Y-A + 34: Mice receiving 80 µg of IgY-A + 34 after challenge with the bacteria.

The LD_50_ of *A. baumannii* AbI101 was determined at 2.04 × 10^7^ CFU. Eye infection was scored based on the severity of the infection. A higher score shows more severe infection. On day 8 post-infection, an average score of eye infection for various groups was in descending order as follow: 2IgY-A + 34 (received 80 µg IgY) = IgY-A + 34 > positive control (received only bacteria) > IgY-C > IgY-A > IgY-34 = IgY-IWC (Supplementary Fig. S2). The Control group received only IgY, showed no eye infection. On day 8 post-infection, average scores of clinical symptoms were as follows: 2IgY-A + 34 = control, received only bacteria > IgY-A + 34 > IgY-C ≥ IgY-34 > IgY-A > IgY-IWC (Supplementary Fig. S3). The Control group received only IgY showed no clinical signs. Based on survival monitoring, all control mice died within 5 days post-infection. A surprising result was the death of all 2IgY-A + 34 group mice within 4 days post-infection. The survival rate of IgY-A + 34, IgY-C, IgY-34, IgY-A, and IgY-IWC groups was 25%, 50%, 50%, 75% and 75% respectively (Fig. 6). After plating the homogenized spleen samples, no colony was seen on plates of IgY-C, IgY-A, or IgY-IWC groups. However, there were 24 and 34 viable bacterial colonies per gram spleen of Y-34 and IgY-A + 34 groups respectively. The viable bacteria calculated in the lungs from various groups were as follow: IgY-C: 3.5 × 10^6^ CFU, IgY-A: 2.02 × 10^7^ CFU, IgY-34: 2.1 × 10^3^ CFU and IgY-A + 34: 2.1 × 10^7^ CFU, and IgY-IWC: 1.8 × 10^5^ CFU.

## Discussion

Our previous publication was the first report on the protective effects of nasal administration of specific IgYs against *A. baumannii*^19^. However, the therapeutic (post-challenge) effects of these antibodies are yet to be addressed. Synergic effect of OmpA and Omp34 had been observed regarding their ability to trigger antibody responses. In contrast, mice received specific IgYs, raised against combination of OmpA and Omp34, showed ADE of *A. baumannii* infection. Although a hypothesis had been suggested for these phenomena, the reason behind these observations remains to be elucidated. Since no additional studies had been carried out to explore these contradictory phenomena (synergic effect and ADE), the current study was conducted. In addition to evaluation of therapeutic (post-challenge) potential of specific IgYs (developed against rOmpA, rOmp34, and inactivated whole-cell of *A. baumannii* in a murine pneumonia model), the in silico analyses, immunoassays and a part of challenges are designed to explore details and to propose reasons for the observed phenomena. The synergic effect of OmpA and Omp34 from antibody triggering point of view was attributed to the existence of similar epitopes in OmpA and Omp34^19^. Similar peptides within OmpA and Omp34 sequences, revealed by in silico analyses, justify the synergic effect of these antigens in the elicitation of antibodies. The cross-reactivity of IgY-34 with OmpA, and IgY-A with Omp34 in the ELISA assay confirmed the existence of similar epitopes within the two OMPs. The higher cross-reactivity of IgY-34 with rOmpA in comparison to cross-reactivity of IgY-A with rOmp34 implies that similar epitopes in the context of the Omp34 sequence are probably more immunodominant than those presented in the context of OmpA sequence.

ELISA results of IgY-A + 34 and IgY-mix proved obvious synergic effects of rOmpA and rOmp34 in terms of triggering specific antibodies. These results revealed that co-administration of these antigens could result in an increased number of similar epitopes followed by an increase in antibody response against these epitopes. This finding is in agreement with Lin et al.^22^ who showed that the escalation of *A. baumannii* rOmpA antigen dose could alter immune polarization and immunodominant epitopes. The escalation of rOmpA antigen dose could enhance epitope spreading and Type 2 immune response. The reactivity of IgY-IWC with rOmpA and rOmp34 in the western blotting as well as ELISA indicates that these two proteins are expressed under in vitro culture conditions. This result is in line with the previous immuno-proteomic study conducted by Fajardo Bonin et al.^21^. OmpA has higher antigenicity than Omp34^19^. Moreover, OmpA is the most abundant OMP in *A. baumannii*; and its expression is significantly higher than Omp34^32^. So, the approximately twofold higher absorbance of IgY-IWC against rOmpA than IgY-IWC against rOmp34 in the ELISA could be due to higher antigenicity of rOmpA than rOmp34 as well as its higher number on the bacterial surface. The whole-cell ELISA results with IgY-A and IgY-34 also in support of the above. In addition to higher titers of specific anti-rOmpA and anti-rOmp34 IgYs in IgY-A + 34, the observed higher absorbance of IgY-A + 34 against whole cell antigen could be attributed to co-existence of exposed OmpA and Omp34 epitopes on the *A. baumannii* surface.

The specific anti-rOmpA and anti-rOmp34 IgYs raised against these OMPs, were purified and administered in denatured condition. Owing to perturbation of their structures and consequently their conformational epitopes, only IgYs raised against their exposed linear B-cell epitopes could theoretically recognize OmpA and Omp34 on the surface of *A. baumannii* (native proteins) and also could confer protection against *A. baumannii*^19^. In the pilot mice challenge, since no significant difference existed between the death time of mice receiving 40 or 100 µg of IgYs, the lower dose (40 µg) was selected for administration in further challenges. Cyclophosphamide-induced neutropenic mice are a suitable model to study *A. baumannii* pneumonia since this model is mimicking the opportunistic nature of this pathogen in the hospital setting^33^. OmpA plays pivotal roles in *A. baumannii* pathogenicity and its adherence to the epithelial cells^20^. This is the most abundant OMP in *A. buamannii*. So, higher protection observed in neutropenic mice receiving IgY-A is reasonable.

A surprising result in our recent study was ADE caused by IgY-A + 34^19^ which also occurred in the current study. Since both antigens are presented on *A. baumannii*, and also in concordance with the ELISA results, higher protection was expected as observed for OmpA, OmpC, and OmpF in *Salmonella enterica*^34^. In addition to the previous study^19^, a recently published report on ADE of *A. baumannii* infection^28^ evaluates the therapeutic effect of a monoclonal antibody (mAb) against the K2 capsular polysaccharide of *A. baumannii*. Surprisingly, not only no protection was achieved; but also the mAb enhanced the infection of a clinical strain of *A. baumannii* in the mouse pneumonia model with increased mice mortality and bacterial burden in the lungs, spleens, and blood. The phenomenon was attributed to the interaction of the mAb with capsule shed as decoys. Although some possibilities expressed for the phenomenon, the mechanisms explaining observed ADE of infection for the pathogen have not been completely understood^28^. ADE had also been reported for lethal toxin activity of *Bacillus anthracis* via a subset of mAbs developed against its protective antigen. It seems that the observed ADE is to some extent epitope-specific^35^ and a subset of mAbs developed against this antigen was toxin-neutralizing^35,36^. The observed ADE for the lethal activity of *Bacillus anthracis* toxin is Fcγ receptor-dependent^36^. This mechanism has also been suggested as a possibility for ADE of *A. baumannii* infection caused by mAbs directed against its K2 capsular polysaccharide^28^. However, this mechanism does not explain ADE observed in our studies; because IgY could not interact with the mammalian Fc receptor^13,37^. Hence, the existence of murine peptides as decoys for IgYs raised against epitopes, responsible for the observed synergic effect and cross-reactivity in ELISA, is more reasonable. The in silico analyses demonstrated that the majority of similar peptides particularly in OmpA are topologically inaccessible. Hence, if existence of decoys (murine peptides) is not a true assumption, higher doses of IgY-A + 34 is anticipated to compensate for idle specific IgYs generated against topologically inaccessible epitopes of OmpA and Omp34. Contradictorily, death of all mice receiving higher amount (80 µg) of IgY-A + 34 is supporting existence of murine peptide as decoys as shown by in silico analyses. Two epitopes of OmpA shared similarity with some proteins in mice. Some of these proteins are among immune system molecules. Some idle antibodies raised against the peptides topologically inaccessible could bind to some host immune system molecules such as CD180 antigen. One advantage of IgY is its ability to be triggered against highly conserved mammalian epitopes due to the genetic differences between chicken and mammals^38^. Although this advantage is favorable in diagnostic applications, it could be considered as a disadvantage in therapeutics. This issue may be considered from the safety point of view for therapeutic applications of IgY. Although the in silico results revealed existence of similar linear epitopes in mouse, further studies are needed to assess the immune response using the murine pneumonia model. This will unveil existence of any direct link between these epitopes and the antibody-dependent enhancement of the disease. Biophysical studies would add to dimensions of the molecular basis of cross reactivity in term of interaction of IgYs with OmpA and Omp34. In conclusion, the specific IgY antibodies raised against OmpA, Omp34, and inactivated the whole-cell of *A. baumannii* showed a therapeutic effect in the murine pneumonia model. Amongst, IgYs raised against OmpA or inactivated the whole-cell of *A. baumannii* had the highest protective effect in neutropenic mice. So, IgY could be employed as a novel effective natural biotherapeutics against pneumonia caused by *A. baumannii*. However, the ADE observed in mice receiving IgY-A + 34 highlights the importance of determination of epitopes responsible for this phenomenon. Designed antigens possessing no epitope responsible for the ADE could elicit safe protective antibodies.

## Supplementary information

Supplementary Information

## References

[1] Perez F, Bonomo RA (2014). Vaccines for *Acinetobacter baumannii*: thinking “out of the box”. Vaccine.

[2] Tacconelli E (2018). Discovery, research, and development of new antibiotics: the WHO priority list of antibiotic-resistant bacteria and tuberculosis. Lancet. Infect. Dis.

[3] Huang W (2016). Immunization with a 22-kDa outer membrane protein elicits protective immunity to multidrug-resistant *Acinetobacter baumannii*. Sci. Rep..

[4] Ballouz T (2017). Risk factors, clinical presentation, and outcome of *Acinetobacter baumannii* bacteremia. Front. Cell. Infect. Microbiol..

[5] Neshani, A. *et al.* Antimicrobial peptides as a promising treatment option against Acinetobacter baumannii infections. *Microbial Pathogenesis*, 104238 (2020). 10.1016/j.micpath.2020.104238 32387392

[6] Bassetti, M., Labate, L., Russo, C., Vena, A. & Giacobbe, D. R. Therapeutic options for difficult-to-treat A cinetobacter baumannii infections: a 2020 perspective. *Expert Opinion on Pharmacotherapy*, 1–11 (2020). 10.1080/14656566.2020.1817386 32915685

[7] Isler, B., Doi, Y., Bonomo, R. A. & Paterson, D. L. New treatment options against carbapenem-resistant Acinetobacter baumannii infections. *Antimicrobial agents and chemotherapy* **63** (2019). 10.1128/AAC.01110-18 PMC6325237 30323035

[8] Ahmad TA, Tawfik DM, Sheweita SA, Haroun M, El-Sayed LH (2016). Development of immunization trials against *Acinetobacter baumannii*. Trials Vaccinol..

[9] McConnell MJ, Pachón J (2010). Active and passive immunization against Acinetobacter baumannii using an inactivated whole cell vaccine. Vaccine.

[10] Luo G (2012). Active and passive immunization protects against lethal, extreme drug resistant-*Acinetobacter baumannii* infection. PLoS ONE.

[11] KuoLee R (2015). Intranasal immunization protects against *Acinetobacter baumannii*-associated pneumonia in mice. Vaccine.

[12] Huang W (2015). OmpW is a potential target for eliciting protective immunity against *Acinetobacter baumannii* infections. Vaccine.

[13] Asadi-Ghalehni M (2015). Cancer immunotherapy by a recombinant phage vaccine displaying EGFR mimotope: an in vivo study. Immunopharmacol. Immunotoxicol..

[14] Müller S, Schubert A, Zajac J, Dyck T, Oelkrug C (2015). IgY antibodies in human nutrition for disease prevention. Nutr. J..

[15] Kollberg H (2003). Oral administration of specific yolk antibodies (IgY) may prevent Pseudomonas aeruginosa infections in patients with cystic fibrosis: a phase I feasibility study. Pediatr. Pulmonol..

[16] Thomsen K (2015). Anti-Pseudomonas aeruginosa IgY antibodies induce specific bacterial aggregation and internalization in human polymorphonuclear neutrophils. Infect. Immun..

[17] Thomsen K (2016). Anti-Pseudomonas aeruginosa IgY antibodies promote bacterial opsonization and augment the phagocytic activity of polymorphonuclear neutrophils. Hum. Vaccines Immunother..

[18] Thomsen K (2016). Anti-Pseudomonas aeruginosa IgY antibodies augment bacterial clearance in a murine pneumonia model. J. Cyst. Fibros..

[19] Jahangiri A (2019). Specific egg yolk antibodies (IgY) confer protection against *Acinetobacter baumannii* in a murine pneumonia model. J. Appl. Microbiol..

[20] Smani Y, McConnell MJ, Pachón J (2012). Role of fibronectin in the adhesion of *Acinetobacter baumannii* to host cells. PLoS ONE.

[21] Fajardo Bonin, R. *et al.* Identification of immunogenic proteins of the bacterium Acinetobacter baumannii using a proteomic approach. *PROTEOMICS Clin. Appl.* **8**, 916–923 (2014). 10.1002/prca.201300133 24899143

[22] Lin L (2013). *Acinetobacter baumannii* rOmpA vaccine dose alters immune polarization and immunodominant epitopes. Vaccine.

[23] Islam AHMS, Singh K-KB, Ismail A (2011). Demonstration of an outer membrane protein that is antigenically specific for *Acinetobacter baumannii*. Diagn. Microbiol. Infect. Dis..

[24] Weber BS, Kinsella RL, Harding CM, Feldman MF (2017). The secrets of Acinetobacter secretion. Trends Microbiol..

[25] Chen W (2015). Current advances and challenges in the development of Acinetobacter vaccines. Human vaccines & immunotherapeutics.

[26] Jahangiri A, Rasooli I, Owlia P, Fooladi AAI, Salimian J (2018). An integrative in silico approach to the structure of Omp33-36 in *Acinetobacter baumannii*. Comput. Biol. Chem..

[27] Jahangiri A, Rasooli I, Owlia P, Fooladi AAI, Salimian J (2018). Highly conserved exposed immunogenic peptides of Omp34 against *Acinetobacter baumannii*: an innovative approach. J. Microbiol. Methods.

[28] Wang-Lin, S. X. *et al.* Antibody Dependent Enhancement of Acinetobacter baumannii Infection in a Mouse Pneumonia Model. *J. Pharmacol. Exp. Therapeut.* (2019). 10.1124/jpet.118.253617 30606761

[29] Jahangiri A, Rasooli I, Owlia P, Fooladi AAI, Salimian J (2017). In silico design of an immunogen against Acinetobacter baumannii based on a novel model for native structure of Outer membrane protein A. Microb. Pathog..

[30] Pirovano W, Feenstra KA, Heringa J (2008). PRALINE^TM^: a strategy for improved multiple alignment of transmembrane proteins. Bioinformatics.

[31] Lieberman HR (1983). Estimating LD50 using the probit technique: a basic computer program. Drug Chem. Toxicol..

[32] Bhamidimarri, S. P. *et al.* A multidisciplinary approach towards identification of novel antibiotic scaffolds for Acinetobacter baumannii. bioRxiv, 306035 (2018). 10.1016/j.str.2018.10.021 30554842

[33] Manepalli S (2013). Characterization of a cyclophosphamide-induced murine model of immunosuppression to study Acinetobacter baumannii pathogenesis. J. Med. Microbiol..

[34] Toobak H (2013). Immune response variations to *Salmonella enterica* serovar Typhi recombinant porin proteins in mice. Biologicals.

[35] Little SF, Webster WM, Fisher DE (2011). Monoclonal antibodies directed against protective antigen of *Bacillus anthracis* enhance lethal toxin activity in vivo. FEMS Immunol. Med. Microbiol..

[36] Mohamed N (2004). Enhancement of anthrax lethal toxin cytotoxicity: a subset of monoclonal antibodies against protective antigen increases lethal toxin-mediated killing of murine macrophages. Infect. Immun..

[37] Carlander D, Kollberg H, Wejåker P-E, Larsson A (2000). Peroral immunotheraphy with yolk antibodies for the prevention and treatment of enteric infections. Immunol. Res..

[38] Kovacs-Nolan J, Mine Y (2012). Egg yolk antibodies for passive immunity. Ann. Rev. Food Sci. Technol..

