# Supplementary material for: Specific egg yolk immunoglobulin as a promising non-antibiotic biotherapeutic product against Acinetobacter baumannii pneumonia infection
Source: Sci Rep. 2021 Jan 21;11:1914. doi: 10.1038/s41598-021-81356-8 (PMC7820402; doi:10.1038/s41598-021-81356-8)
Supplement: Supplementary file 1 — Supplementary Information [file 41598_2021_81356_MOESM1_ESM.docx]

# **Specific egg yolk immunoglobulin as a promising non-antibiotic biotherapeutic product against *Acinetobacter baumannii* pneumonia infection**

Abolfazl Jahangiri^1,2^, Parviz Owlia^3,4^, Iraj Rasooli^1,3^^[[1]](#footnote-1)^*, Jafar Salimian^5^, Ehsan Derakhshanifar^6^, Zahra Aghajani^1^, Sajad Abdollahi^7^, Saeed Khalili^8^, Daryush Talei^9^, Elham Darzi Eslam^1^

1- Department of biology, Shahed University, Tehran-Iran.

2- Applied Microbiology Research Center, Systems Biology and Poisonings Institute, Baqiyatallah University of Medical Sciences, Tehran, Iran

3- Molecular Microbiology Research Center and Department of Biology, Shahed University, Tehran-Iran

4- Department of Microbiology, Shahed University Faculty of Medical Sciences, Tehran, Iran

5- Chemical Injuries Research Center, Systems Biology and Poisonings Institute, Baqiyatallah University of Medical Sciences, Tehran, Iran

6- Ph.D. Student of Medical Biotechnology, Hamadan University of Medical Sciences, Hamadan, Iran

7- Behbahan Khatam Alanbia University of Technology

8- Department of Biology Sciences, Shahid Rajaee Teacher Training University Tehran, Iran

9- Medicinal Plants Research Center, Shahed University, Tehran, Iran

**Supplementary data**

**
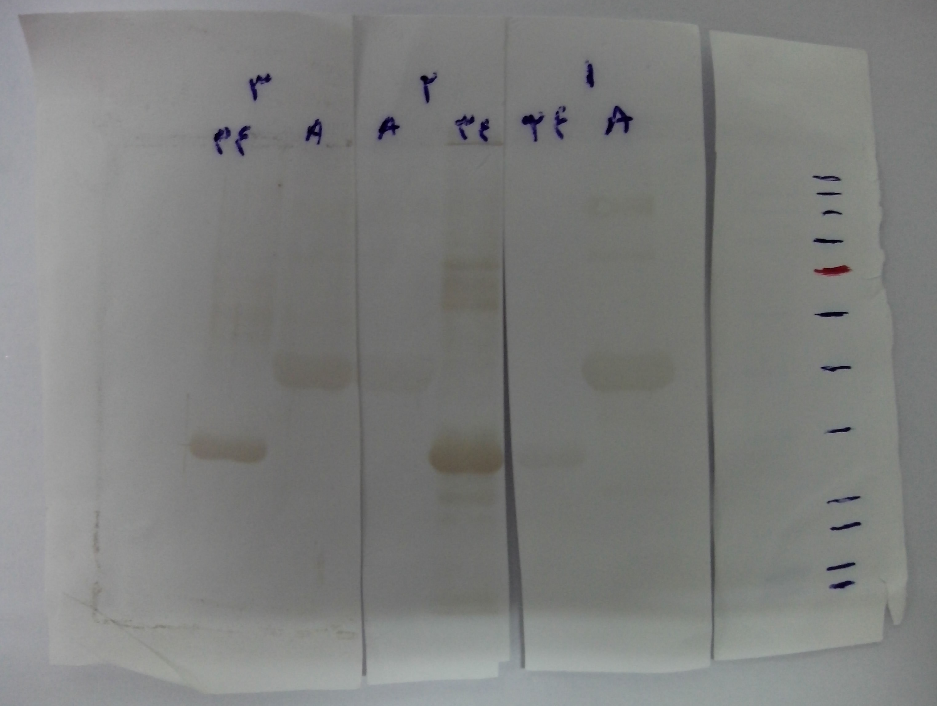
**


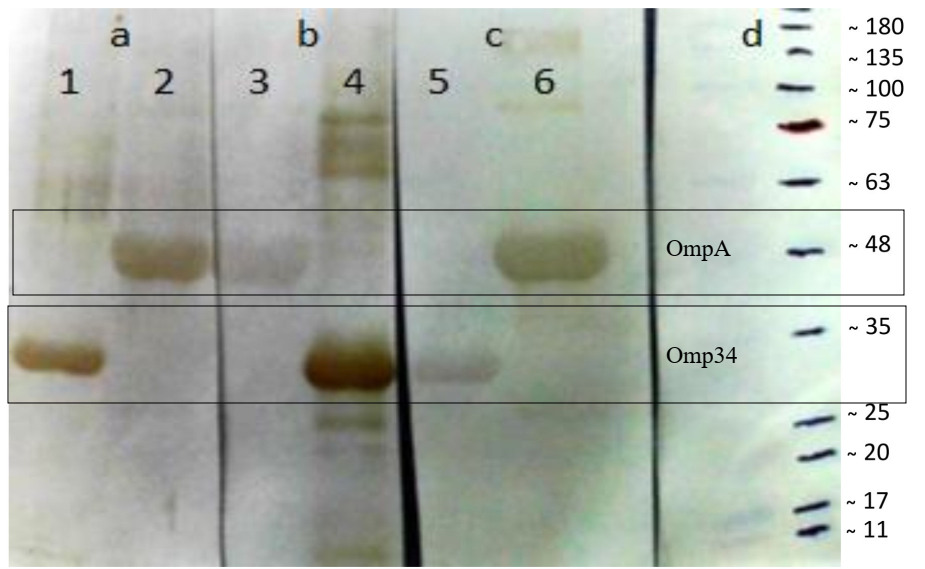


**Supplementary Figure S1.** Western blotting with specific IgYs

(**a**) strip incubated with IgY-IWC, (**b**) strip incubated with IgY-34, (**c**) strip incubated with IgY-A, (**d**) protein weight marker; Lanes 1, 4 and 5: rOmp34, Lanes 2, 3 and 6: rOmpA.

The depicted parts are strips from an individual blotting process. However, since each strip is treated with a unique antibody, they had to be processed separately. The image is cropped by Microsoft Office 2010. Changing of its brightness and contrast was equally done for all parts by Microsoft Office 2010. The texts were embedded by Microsoft PowerPoint 2010. No further changes were exerted to the original Figure.


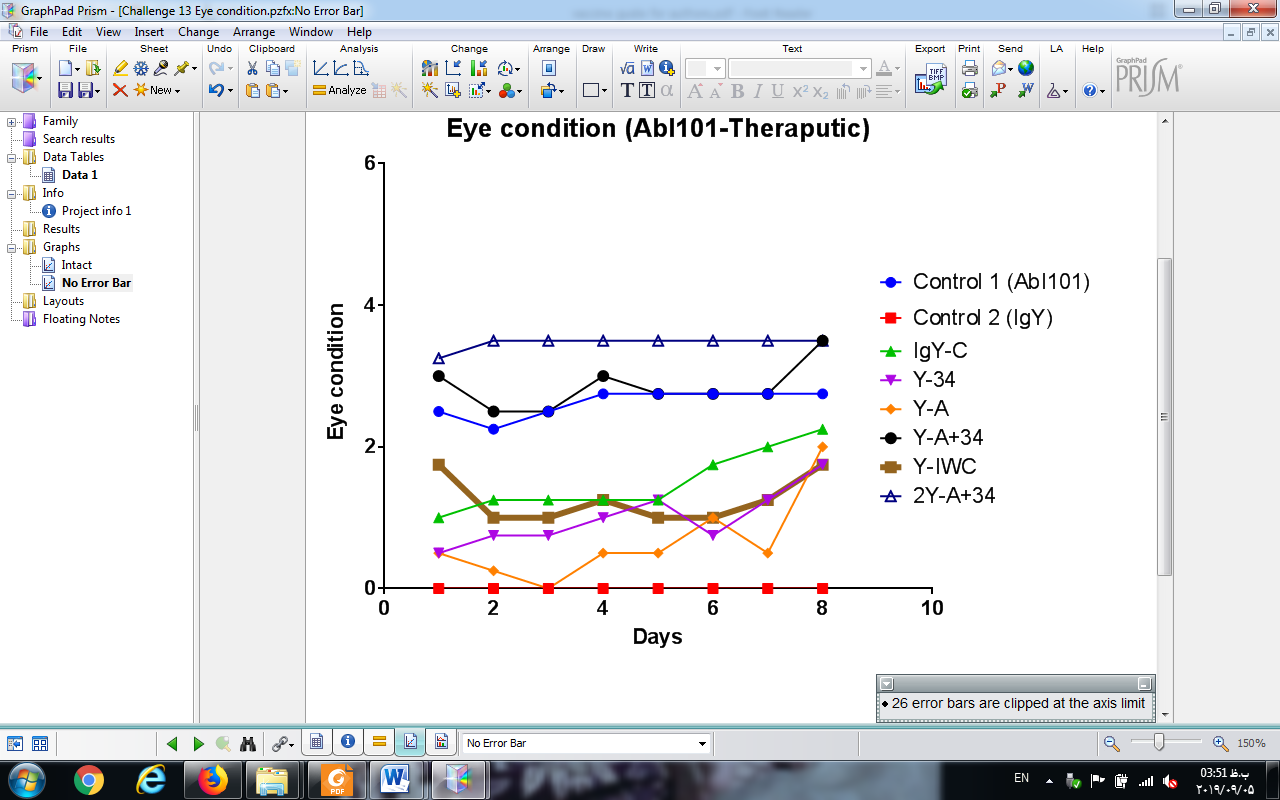


**Supplementary Figure S2.** Average eye condition of mice groups receiving therapeutic mode of IgY 4 hours post infection with *A. baumannii* AbI101. The mice were monitored for 8 days. 0: no sign, 4: closed and infected eye. Control 1: Mice receiving 5.65 × 10^8^ CFU of *A. baumannii* AbI101, Control 2: receiving 40 µg of IgY,Y-A+34: receiving 40 µg of IgY-A+34 after challenge with the bacteria, Y-C: receiving 40 µg of IgY-C after challenge with the bacteria, Y-34: receiving 40 µg of IgY-34 after challenge with the bacteria, Y-IWC: receiving 40 µg of IgY-IWC after challenge with the bacteria, 2Y-A+34: receiving 80 µg of IgY-A+34 after challenge with the bacteria, IgY-A: receiving 40 µg of IgY-A after challenge with the bacteria.


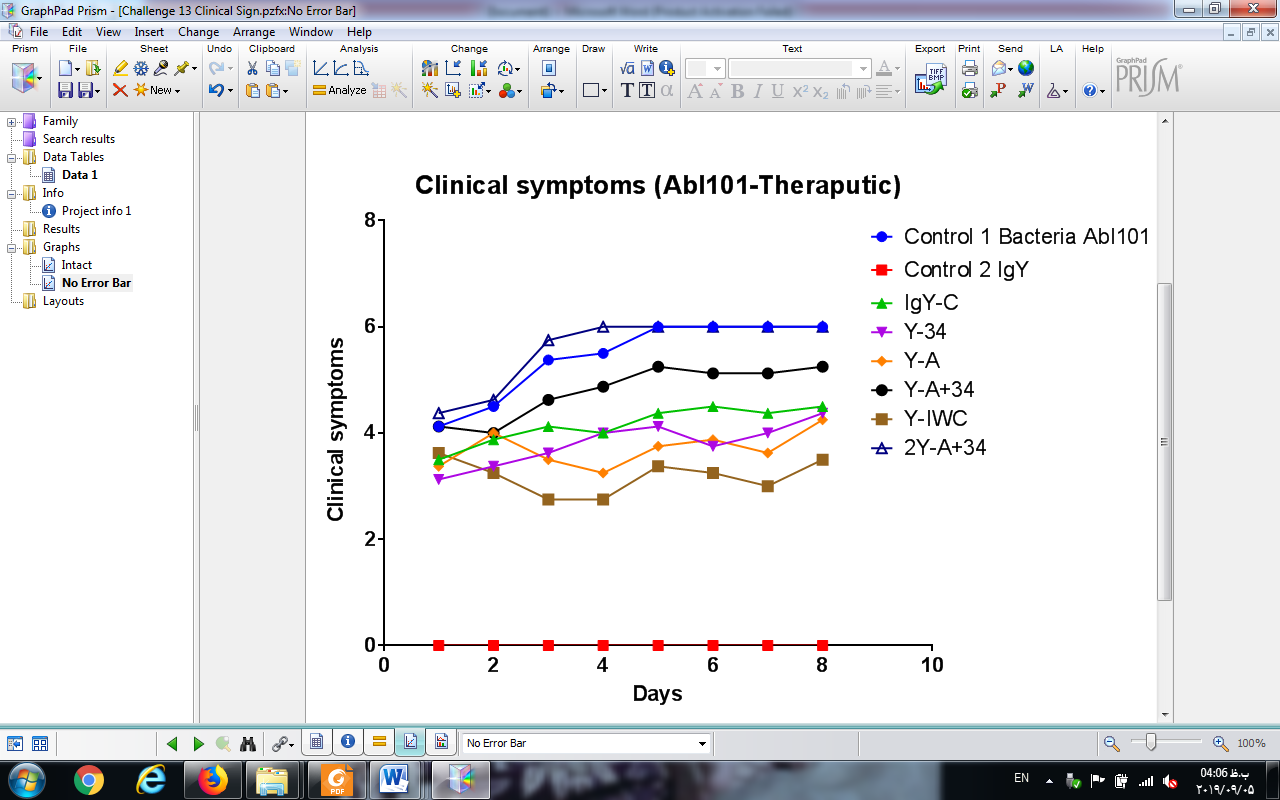


**Supplementary Figure S3.** Average clinical symptoms of mice groups receiving therapeutic mode of IgY 4 hours post infection with *A. baumannii* AbI101. The mice were monitored for 8 days. 0: no sign, 6: dead. Control 1: Mice receiving 5.65 × 10^8^ CFU of *A. baumannii* AbI101, Control 2: receiving 40 µg of IgY,Y-A+34: receiving 40 µg of IgY-A+34 after challenge with the bacteria, Y-C: receiving 40 µg of IgY-C after challenge with the bacteria, Y-34: receiving 40 µg of IgY-34 after challenge with the bacteria, Y-IWC: receiving 40 µg of IgY-IWC after challenge with the bacteria, 2Y-A+34: receiving 80 µg of IgY-A+34 after challenge with the bacteria, IgY-A: receiving 40 µg of IgY-A after challenge with the bacteria.

1. *Corresponding author, Biology Department, Shahed Unversity, Tehran-Qom Express way, Tehran-3319118651, Iran. Tel; +98 (21)51212200, Fax +98 (21)51212201, Email: [rasooli@shahed.ac.ir](mailto:rasooli@shahed.ac.ir) [↑](#footnote-ref-1)
